# Stability study of docetaxel solution (0.9%, saline) using Non-PVC and PVC tubes for intravenous administration

**DOI:** 10.1186/s40824-014-0023-x

**Published:** 2015-01-28

**Authors:** Kang Hoon Park, Dong June Chung

**Affiliations:** Department of Polymer Science & Engineering, Sungkyunkwan University, Suwon, 440-746 Korea

**Keywords:** Docetaxel, Infusion tube, Non-PVC, Di-2-ethylhexyl phthalate(DEHP), Polysorbate 80

## Abstract

**Background:**

Di-2-ethylhexyl phthalate (DEHP) are added to poly(vinyl chloride)(PVC) infusion tubes as a plasticizer to ensure tube flexibility. In addition to previously reported disadvantages of DEHP, released DEHP molecules from PVC tubes can easily interact with surfactants in anticancer drug solutions (i.e., polysorbate 80 for Taxotere®-Inj) and reduce the solubility of docetaxel in aqueous solution during anticancer drug administration.

**Results:**

In this study, we investigated the *in vitro* stability of docetaxel in a 0.9% saline solution under an intravenous administration condition using a PVC tube (high DEHP content) and non-PVC infused tube.

**Conclusion:**

The docetaxel solution circulating through the non-PVC tube had better solution stability than through the PVC tube(high DEHP content).

## Background

Poly(vinyl chloride) (PVC) is a widely used material for intravenous infusion tubes for administration of drugs in clinical practice and blood bags for blood storage after blood-gathering. Most intravenous infusion PVC tubes contain di-2-ethylhexyl phthalate (DEHP) as a plasticizer to provide flexibility of the infusion tube.

Many reports exist regarding DEHP’s toxic effects released from the PVC tube during intravenous infusion of aqueous drug solutions [1,2]. Leached DEHP affects human metabolism [3,4] and can decrease the medicinal efficacy owing to drug adsorption on the inner surface of the PVC infusion tube [5]. In addition to the drug adsorption effect due to DEHP release, surfactants(such as polysorbate 80 [Tween®80] and polyethoxylated castor oil [Cremophor®EL]) which are meant enhance anticancer drug solubility in the solvent, can interact with leached DEHP from the infusion tube [6]. The plasticizer can affect the surfactants efficacy and reduce the solubility of the lyophilic anticancer drug in the aqueous solution, thus the precipitated anticancer drug (which finally occur concentration difference in anticancer drug solution) may not retain enough of its original pharmaceutical efficacy.

In this study, to confirm the plasticizer effect in chemotherapy using clinical infusion tubes, we investigated the stability of docetaxel solution (0.9 wt% in saline) circulating through commercial PVC (high DEHP content), manufactured PVC (low DEHP content) and non-PVC (polyolefin) infusion tubes.

## Methods

### Materials

The docetaxel solution (anticancer agent, Taxotere®-Inj) was purchased from Sanofi-Aventis Korea Co. Ltd. (Seoul, Korea). Commercial PVC infusion tubes (DEHP content: 25 wt%) were purchased from Becton Dickinson Co. Ltd. (Franklin Lakes, NJ, USA). Another PVC (DEHP content: 10 wt%) tube and non-PVC infusion tubes as control group were manufactured by Polyscientech Co. Ltd. (Hwaseong, Korea) using commercial PVC resin (Hanwha Chemical, Co. Ltd., Seoul, Korea) and polyolefin composite as the polyethylene (PE) elastomer/polypropylene(PP) elastomer/polybutadiene(PB) blend(25/50/25 weight ratio), respectively. The PE elastomer (Infuse®, melt index, g/min, 190°C, 2.16 Kg; 5.0) and PP elastomers (Vistamaxx®, melt index, g/min, 190°C, 2.16 Kg; 3.0) were obtained from Dow Chemical Co. Ltd. (Midland, MI, USA) and Exxon-Mobil Chemical Co. Ltd. (Houston, TX, USA), respectively. The PB elastomer (syndiotactic 1,2- polybutadien, melt index, g/min, 150°C, 21.2 N; 3.0) was obtained from JSR Corporation (Japan Synthetic Rubber Corporation, Tokyo, Japan).

The three tubes were adapted for comparison of the time-dependent solution stability of docetaxel during the infusion period (approximately 1 hr).

### Methods

As shown in Table 1, docetaxel solution for circulation was prepared as follows: 20 mg of docetaxel (1 mL Taxotere®-Inj solution containing polysorbate 80) was dissolved in 1 mL of dehydrated ethanol. The solution was dissolved in 100 mL of 0.9 wt% saline solution as the standard according to the manufacturer’s instructions (Sanofi-Aventis Korea).

**Table 1:** **Composition of the Taxotere®-Inj anticancer drug**

Docetaxel (anhydrous)	20 mg
Polysorbate 80	0.54 g
Dehydrated ethyl alcohol	0.395 g (0.5 mL)
Total volume	1 mL

**Figure 1:**
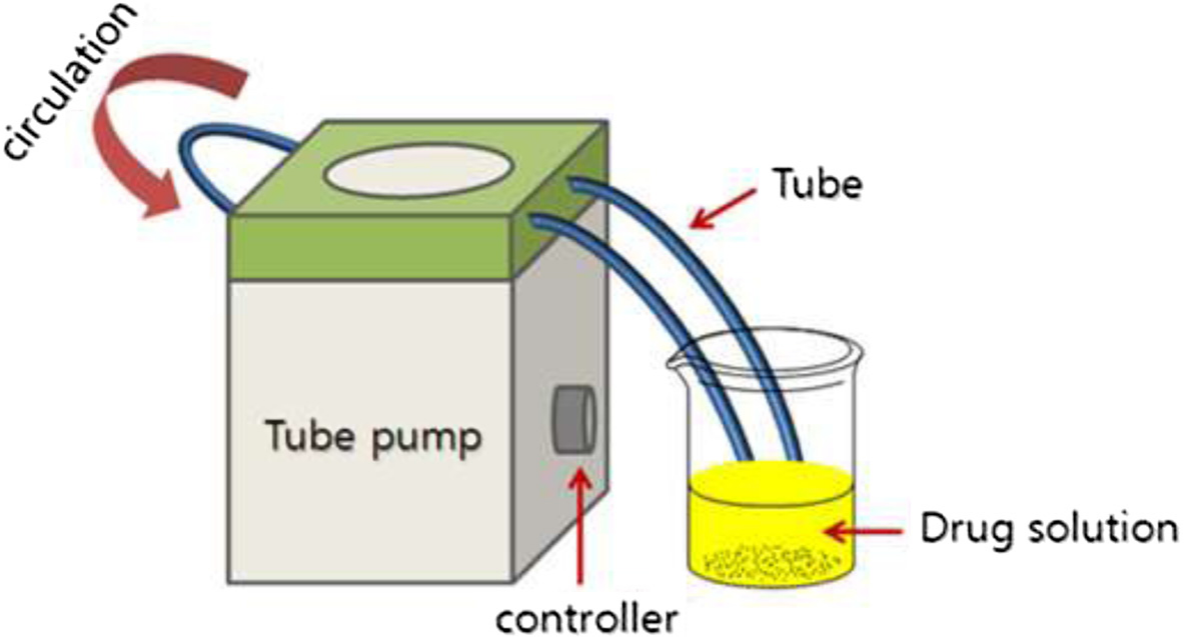
**Schematic diagram of the circulation experiment.**

The infusion tube length (commercial PVC infusion tube; 85 cm, non-PVC tube and manufactured PVC (low DEHP content) tube; 100 cm) was determined with the invariable inner surface area (75 cm^2^) based on the tubes having different inner diameters (commercial PVC tube, 2.8 mm; non-PVC tube and manufactured PVC tube, 2.4 mm). The circulation experiment was conducted using a micro tubing pump (EYELA MP-3 N, Tokyo Rikakikai Co. Ltd., Tokyo, Japan) and three types of infusion tubes were mounted on the tubing pump (Figure 1).

The circulating rate of the drug solution through the three types of tubes was maintained between 1.2 - 3.0 mL/min to maintain a constant circulating volume considering the different inner diameters of the infusion tubes. The stability of drug solution was evaluated by measuring the visible light (600 nm) transmittance changes of the circulating drug solution at predetermined time intervals (every 10 min).

And then concentration differences of docetaxel solution after circulation was evaluated by measuring the high-performance liquid chromatography (HPLC). The HPLC system was equipped with Waters 1525 binary HPLC pump, Waters 2487 dual λ absorbance detector. HPLC separation was achieved on a Gemini 5 μ C18 column (150 × 4.60 mm, 5micron) (Phenomenex®). Column effluent was monitored at 227 nm. The mobile phase was a mixture of distilled/deionized water and acetonitrile (50:50 v/v) with 1 ml/min flow rate [7]. Standard solution of docetaxel is prepared by dissolving 20 mg of docetaxel in 100 ml of 0.9 wt% saline solution. And standard solutions were prepared in the range of 40-200 μg/ml. The retention time of docetaxel was about 8.0 min.

## Results and discussion

**Figure 2:**
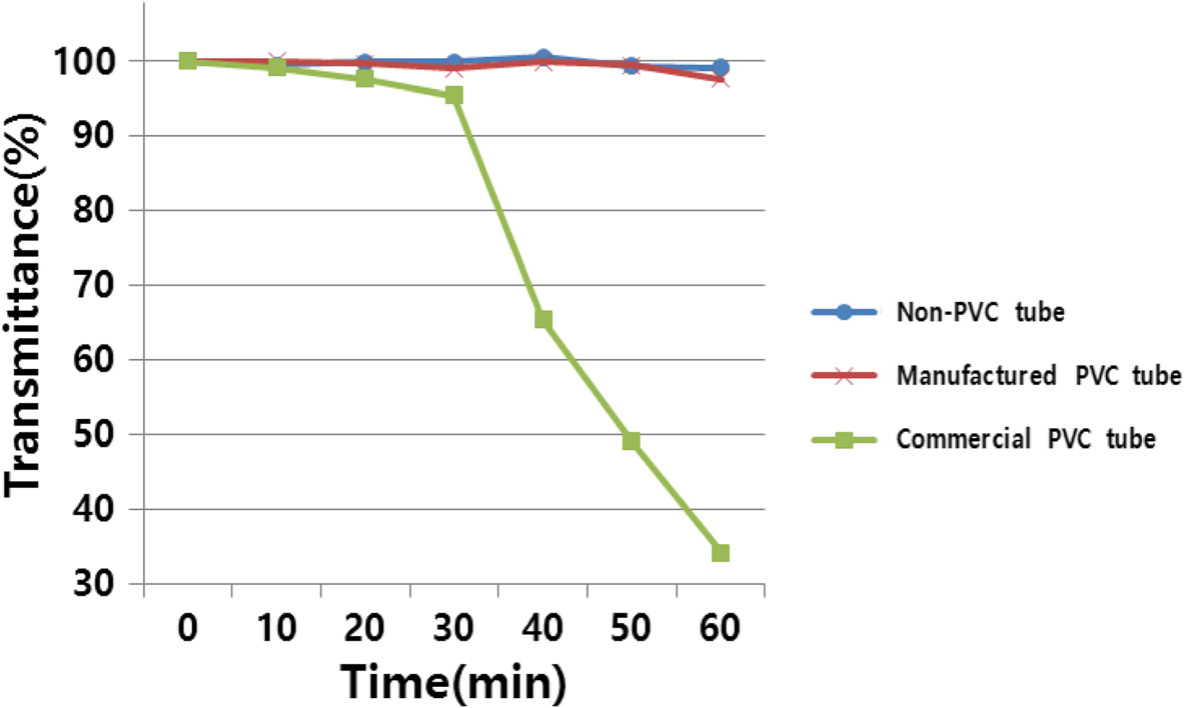
**Visible light (600 nm) transmittance change of docetaxel solution during the circulation experiment using commercial PVC(high DEHP content), manufactured PVC (low DEHP content) and non-PVC infusion tubes.**

The visible light transmittance changes at 600 nm of docetaxel solution every 10 min during 1 hr of circulation is shown in Figure 2.

**Figure 3:**
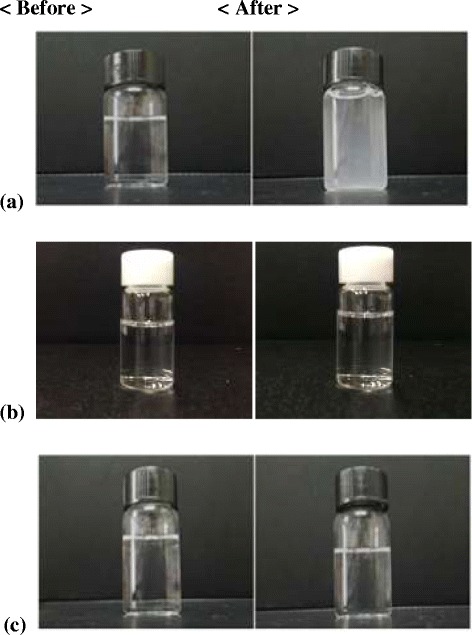
**Stability differences of docetaxel solution after circulation; (a) commercial PVC tube, (b) manufactured PVC tube, (c) non-PVC tube.** Left: before circulation, right: after 1 hr circulation.

According to the instructions from the docetaxel solution manufacturing company, infusion time of docetaxel solution in the clinical field is within 1 hr, which was chosen as the entire circulation time for the drug solution. The docetaxel solution circulating through the commercial PVC tube showed a significant decrease in transmittance after 30 min of circulation and the transmittance decreased to less than 50% of the original value after 50 min. The anticancer drug (docetaxel) precipitated as particles in the 0.9 wt% saline solution after 1 hr circulation. Eventually, the docetaxel solution passing through the PVC tube became hazy due to the precipitation (Figure 3a).

This phenomenon can be explained by the released DEPH from the commercial PVC infusion tube during the circulation process contacting the surfactant molecules (polysorbate 80 in Taxotere®-Inj solution) and readily forming complexes through physical interactions [8]. Therefore, the surfactant content in the Taxotere®-Inj solution was decreased and the docetaxel solubility in the Taxotere®-Inj solution was also reduced. The docetaxel molecules were extracted from the Taxotere®-Inj solution after 1 hr of circulation, indicating that when a PVC infusion tube is used for anticancer therapy in the clinical field, anticancer drug (i.e., docetaxel) molecules are extracted from the Taxotere®-Inj solution during intravenous administration. Therefore, the effective drug concentration can be altered during intravenous administration and the original efficacy of the anticancer drug may not be maintained during chemotherapy.

But, the change in docetaxel solubility passing through the non-PVC tube and manufactured PVC tube for the 1 hr circulation was not observed because that the above mentioned interaction between DEHP and polysorbate 80 was minimized and solution stability was remained (Figures 3b and 3c).

From our previous report [9], the non-PVC tube showed almost identical mechanical properties as the commercial PVC tube in spite of lacking DEHP. And in the case of manufactured PVC tube, the interaction between small amount of released DEHP and surfactants was negligible and docetaxel precipitation was avoided during circulation. In the non-PVC tube and manufactured PVC tube, a decrease in solubility (as shown by lower transmittance) was not observed and drug efficacy during clinical drug injection was preserved.

**Figure 4:**
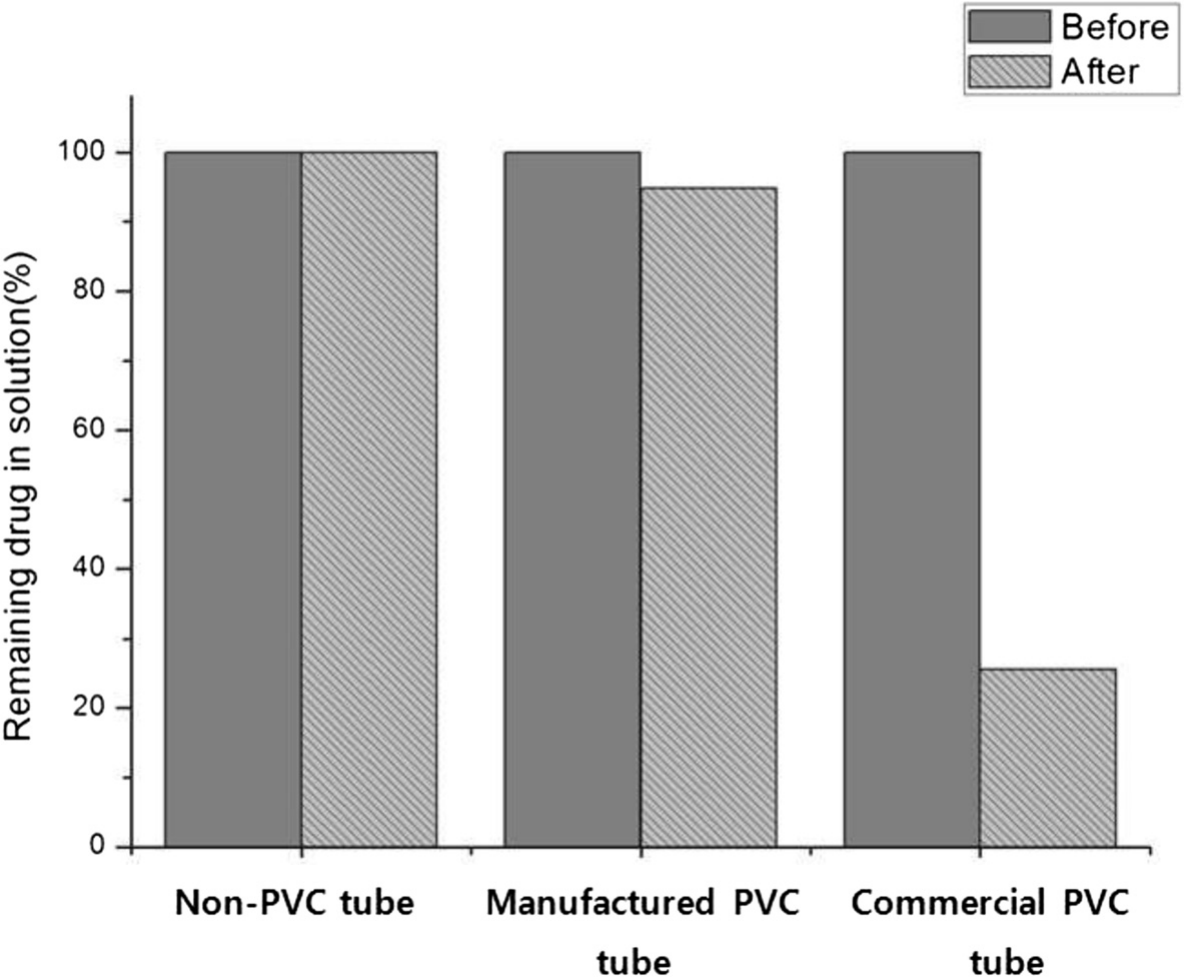
**The concentration differences of docetaxel solution after circulation during 60 min using commercial PVC, manufactured PVC and non-PVC infusion tubes.** Regression equation was calculated Y = 12142X + 10750 and correlation was 0.994. Y is the peak area and X is the concentration in drug (HPLC).

Additionally, the concentration differences of docetaxel solution before/after circulation were evaluated by the HPLC. Non-PVC and manufactured PVC tubes as control group did not show concentration differences. But commercial PVC tube showed noticeable concentration difference up to 75% decrease after circulation (Figure 4).

From pre-mentioned results, manufactured PVC tube may be suitable for infusion tube under the only consideration of negligible interaction with surfactants. But, minimum amount of DEHP was adapted for tube processing, the manufactured PVC tube is not able to recover to original shape after bending (or folding) and is too hard not to use as infusion tube in clinical field.

Therefore, the non-PVC(Olefin) tube is a good candidate for the substitution of PVC infusion tube in chemotherapy.

## Conclusion

The efficacy of an injected anticancer drug was not preserved when commercial PVC infusion tube (containing DEHP as a plasticizer at high content) was used for clinical drug administration. Conversely, a non-PVC infusion tube (polyolefin), which did not contain DEHP, maintained drug efficacy in chemotherapy. Therefore, alternative administration using non-PVC tubes and storage bags should be used widely in medical fields to avoid the risks of DEHP leaching and reduction of drug efficacy.

## Availability of supporting data

**Figure 3:** Stability differences of docetaxelsolution after circulation; (a) commercial PVC tube, (b) manufactured PVC (less DEHP), (c) non-PVC tube. Left: before circulation, right: after 60 min of circulation.

## References

[1] Hanawa T, Endoh N, Suzuki M, Terada K, Ohkuma M, Sakuta K (2005). Release behavior of diethylhexyl phthalate from the polyvinyl-chloride tubing used for intravenous administration and the plasticized PVC membrane. Internat J Pharm.

[2] Demore’ B, Vigneron J, Perrin A, Hoffman MA, Hoffman M (2002). Leaching of diethylhexyl phthalate from polyvinyl chloride bags into intravenous etoposide solution. J Clinical Pharm Therap.

[3] Joel AT, Ted S, Tee G, Michael MC, Mark R (2001). Health risks posed by use of di-2-ethylhexyl phthalate (DEHP) in PVC medical devices: A critical review. Am J Ind Med.

[4] Koch HM, Preuss R, Angerer J (2006). Di(2-ethylhexyl)phthalate (DEHP): human metabolism and internal exposure - an update and latest results. Int J Androl.

[5] Anna T, Gerd W, Rainer B, Frank W (2009). Investigation into the sorption of nitroglycerin and diazepam into PVC tubes and alternative tube materials during application. Int J Pharm.

[6] Takehisa H, Emi M, Kohki A, Masahiko S, Mutsuko T, Kenji K, Toshinobu S, Kazuhiko J, Shin’ichiro N (2000). Investigation of the release behavior of diethylhexyl phthalate from the polyvinyl-chloride tubing for intravenous administration. Int J Pharm.

[7] Kalra N, Nagpal M, Nyola NK (2013). Sensitive high-performance liquid chromatographic method for the determination of taxol category drug in plasma. Int J Pharm Sci.

[8] Yuji H, Fumie S, Tae H, Haruko Y, Chile H, Shunichiro I (2005). Development of a simple method for predicting the levels of di(2-ethylhexyl) phthalate migrated from PVC medical devices into pharmaceutical solutions. Int J Pharm.

[9] Park KH, Park CK, Park J, Jeon SH, Bang SI, Kim JH, Chung DJ (2014). Drug adsorption behavior of Polyolefin infusion tube compared to PVC and PU. Polymer(Korea).

